# Neuromodulation for Pain Management: Evidence of Safety and Efficacy

**DOI:** 10.3390/brainsci16090950

**Published:** 2026-09-07

**Authors:** Andrés Molero-Chamizo, Rafael Tomás Andújar Barroso

**Affiliations:** 1 Psychobiology Area, Department of Clinical and Experimental Psychology, University of Huelva, Campus El Carmen, 21071 Huelva, Spain; 2 Clinical Psychology Area, Department of Clinical and Experimental Psychology, University of Huelva, Campus El Carmen, 21071 Huelva, Spain

## 1. Introduction

Chronic pain is a frequently debilitating symptom of various medical conditions. Its multifactorial organic origin complicates its resolution in many patients, and the search for effective therapeutic options remains a priority in medical research. The three main pain mechanisms—nociceptive, neuropathic, and nociplastic or central sensitization pain—are responsive to different therapeutic approaches that can modulate pain by acting on these mechanisms. Multiple invasive and non-invasive neuromodulation techniques have shown clear potential for impacting these pain mechanisms in clinical practice [1,2]. Complex and diverse clinical trials have analyzed the therapeutic effectiveness of the two main non-invasive neuromodulation methods, magnetic and electrical stimulation, which have also shown a high safety profile [3]. Both transcranial magnetic and electrical stimulation and their variants have proven efficacy and safety in the treatment of various chronic pain conditions, including neuropathic pain, migraine, traumatic and post-surgical neuropathies, headache and occipital neuralgia, chronic low back pain, rheumatological pain, post-traumatic peripheral neuropathic pain, limb pain, fibromyalgia, phantom limb pain, facial pain, brachial plexopathy, trigeminal neuralgia, etc.

The main limitation of these procedures is the interindividual variability in their therapeutic potential and, consequently, their limited prognostic capacity. This variability is primarily due to individual anatomical and physiological differences, as well as differences in the neuromodulation procedure and the stimulation parameters used (number of sessions, total treatment duration, follow-up measures, outcome measures, stimulation intensity and frequency, session duration, target location, etc.). These limitations mean that current neuromodulation procedures have moderate evidence of effectiveness in numerous pain conditions with a good safety profile.

The present Special Issue addresses recent research demonstrating the therapeutic potential and safety of different neuromodulation procedures, with the aim of exploring the latest advances and promoting knowledge and research in the field of pain medicine. This Special Issue includes seven specialized articles, which will be cited in the following section. Without aiming for a detailed description of the work carried out in each of these studies, the purpose of this Editorial is to highlight the objectives of such research in order to generate the necessary interest in neuromodulation procedures and their efficacy and safety in the treatment of chronic pain.

## 2. An Overview of the Articles Published in This Special Issue

The articles published in this Special Issue include a study protocol, two retrospective analysis studies, two clinical trials, one narrative review, and one perspective article. In the first article, Portocarrero-Sánchez et al. describe a prospective intervention protocol with multiple biological, sensory and neuropsychological measures to explore predictors of response to occipital nerve stimulation (ONS) in patients with refractory chronic cluster headache [4]. ONS is a neuromodulation method considered to be minimally invasive, although it involves a surgical implant. Predicting the response to this treatment is one of the current research objectives for improving the safety and efficacy of this therapy. The study design includes a minimum sample of 15 patients. Before the ONS implantation, multiple measures will be recorded (including structural 3T MRI, blood analysis, neuropsychological evaluation, auditory evoked potentials, algometry, and transcutaneous electrical nerve stimulation response). Post-intervention measures will be obtained at 3, 6 and 12 months. Under this protocol, the authors expect to identify predictive biomarkers of ONS response that could improve the process of selecting a priori responder patients.

In the second article, Sethi et al. evaluated the prevalence of medical complications 24 months after tonic permanent percutaneous spinal cord stimulator implantation via retrospective analysis of prospectively collected data [5]. The main objective of this study was to analyze the incidence of the post-operative symptoms of paresthesia and dysesthesia in patients with chronic intractable pain of the trunk and/or limbs, as well as risk factors. The data analysis included 103 eligible patients. The development of unpleasant paresthesia was reported by 27.86% of patients, and 34.95% needed revision due to explantation of the spinal cord stimulation system. Current tobacco use was identified as a significant risk factor. The authors pointed to appropriate waveform selection to reduce the incidence of uncomfortable paresthesia in patients with percutaneous spinal cord stimulator implantation.

Through a retrospective study also published in this Special Issue, Yoo et al. analyzed clinical variables with potential to predict the effectiveness of spinal cord stimulation in the treatment of patients with intractable chronic pain, including complex regional pain and persistent spinal pain syndromes [6]. A 50% reduction in pain according to the numerical rating scale, six months after implantation, was considered a significant effect of the intervention. In a sample of 213 patients, 50.7% were responders after the implantation. However, both the responders and those with no initial response to treatment showed significantly lower pain scores 6 months after the intervention, compared with baseline measures. The proportion of male initial responders was significantly higher than in the negative outcome group (63.0% vs. 50.5%), which points to male gender as a predictor of treatment effectiveness. In contrast, longer pain duration proved to be a negative predictor. No significant adverse events were observed during the six-month follow-up. As the authors suggest, the efficacy of spinal cord stimulation as a treatment for chronic intractable pain appears to be superior in male patients with shorter duration of pain.

In a randomized, double-blind, crossover pilot clinical trial, Portocarrero-Sánchez et al. evaluated the efficacy of repetitive transcranial magnetic stimulation (rTMS) in chronic cluster headache prevention [7]. The efficacy of rTMS (vs. sham) was measured by the number of attacks per week. Secondary outcome measures were tolerability and changes in intensity, duration and use of medication. Three patients received rTMS (10 min sessions per day for 10 consecutive working days) and sham in randomized order, separated by a one-month washout period; five patients received rTMS and six were treated with sham stimulation. The most relevant result was the complete reduction in attacks per week in two patients after 4 days of rTMS, which was reverted after a week of washout, and no significant differences were found between rTMS and sham conditions. No significant differences were found in medication consumption after rTMS and side effects were mild and transient. The authors point to a potential clinical benefit of rTMS in the prevention of refractory chronic cluster headache attacks in selected cases. These effects are transient under the stimulation protocol used.

On the other hand, in a randomized controlled pilot clinical trial, Melián-Ortiz et al. explored the effects of a superficial non-invasive neuromodulation method on different symptoms in post-COVID-19 female patients with at least one year of evolution [8]. This technique uses biphasic microcurrents, with oscillating frequencies (between 1.14 and 14.29 Hz), intensities ranging from 0.1 to 0.9 mA, and low voltage (±3 V), applied over distal nerve terminals of the limbs (six electrodes per limb positioned on the wrists and ankles and directional electrodes). This electrode configuration creates a circulating bioelectric circuit modulating the activity of the autonomic nervous system. In this study, directional electrodes were positioned over the C7 cervical vertebra, and 15 one-hour neuromodulation sessions were carried out (three days a week, on alternate days). In nine patients, real neuromodulation was applied, and in seven, a placebo procedure was used as a control group. Heart rate variability, pain threshold, cortisol level, fatigue, sleep quality, and quality of life were the primary outcome measures. All recruited patients completed the study, with the findings revealing that four measures improved after the intervention in both groups (sleep quality, quality of life, fatigue, and cortisol levels). However, heart rate variability significantly increased in the real neuromodulation group but decreased in the control group, and pain threshold at the C5–C6 vertebral level only significantly improved in the experimental group. The authors conclude that the neuromodulation method used in its study is a safe and well-tolerated therapeutic modality for post-COVID-19 patients, but since no interaction effects between time (pre- vs. post-intervention) and group were found in any measure, the potential specific therapeutic effects of this approach need to be analyzed in large-scale studies.

In the sixth article, Fathi et al. conducted a literature review on the transcranial alternating current stimulation (tACS) effects on pain perception and pain-related brain oscillations in both healthy controls and patients with chronic pain [9]. Nine and five studies comparing tACS and sham effects on experimental pain in healthy participants and different clinical pain conditions, respectively, were included in this review. The most frequently analyzed brain oscillatory activity was the somatosensory alpha rhythm. The authors found considerable variability in the results, which might be attributed to methodological heterogeneity and a lack of knowledge of the underlying mechanisms. The findings range from pain improvements, increases in alpha power band, or changes in the connectivity of sensorimotor and prefrontal cortical regions to non-significant brain or behavioral changes. Specifically, six of the fourteen analyzed studies reported analgesic effects and two observed improvements within subgroups or under specific experimental conditions. According to the authors, methodological heterogeneity and critical differences in individual characteristics may explain this variability in the results. Methodological advances, improvements in protocols, and large-scale studies, as well the use of neuroimaging, electric field modeling and electrophysiological recordings, are proposed to explore in the future the effectiveness of tACS in pain treatment and enhance clinical applicability by personalized approaches.

The last article in this Special Issue develops a perspective on the use of non-invasive brain stimulation (NIBS) procedures and methods, including magnetic and electrical stimulation, in patients with chronic musculoskeletal pain (CMP), exploring important issues such as action mechanisms, patient selection, methodological variations, adverse effects and the safety of interventions [10]. The authors focus on CMP conditions such as myofascial pain syndrome, osteoarthritis and other joint pain syndromes. The authors’ main conclusion refers to the current lack of evidence on the efficacy of NIBS techniques in the treatment of CMP. The authors point to the need for a better understanding of the neural mechanisms underlying each of the different non-invasive neuromodulation techniques, as well as the need to optimize stimulation protocols and identify responder patients. These advances will allow for a determination of the efficacy and safety of these interventions in the treatment of CMP patients.

## 3. Conclusions

This Special Issue has contributed to understanding the current state of knowledge regarding the effectiveness and safety of different non-invasive neuromodulation procedures in treating patients with pain that does not respond to conventional therapies. The methods and medical conditions addressed in the different articles include: ONS to treat refractory chronic cluster headache, tonic percutaneous spinal cord stimulation in the treatment of patients with chronic intractable pain of the trunk and/or limbs and patients with complex regional pain and persistent spinal pain syndromes, rTMS for chronic cluster headache prevention, non-invasive neuromodulation by biphasic microcurrents modulating the autonomic nervous system to treat post-COVID-19 female patients, tACS application to modulate pain perception and pain-related brain oscillations in patients with chronic pain, and the use of magnetic and electric non-invasive brain stimulation methods to treat CMP patients.

The data analyzed in these articles indicate a wide variability in results depending on the specific techniques and protocols used, as well as the individual characteristics of the patients and the type of pain treated. However, the neuromodulation methods analyzed here have generally demonstrated a high safety profile and appear to be promising interventions in pain management with the potential to optimize stimulation parameters and clinical outcomes.
